# Explaining the association between repetition priming and source memory: No evidence for a contribution of recognition or fluency

**DOI:** 10.1177/17470218211008406

**Published:** 2021-04-12

**Authors:** Nicholas Lange, Christopher J Berry

**Affiliations:** 1 Department of Psychology, University of Warwick, Coventry, UK; 2 School of Psychology, University of Plymouth, Plymouth, UK

**Keywords:** Source memory, repetition priming, recognition memory

## Abstract

In a conjoint memory task (measuring repetition priming, recognition memory, and source memory), items recognised as previously studied and receiving correct source decisions also tend to show a greater magnitude of the repetition priming effect. These associations have been explained as arising from a single memory system or signal, rather than multiple distinct ones. In the present work, we examine whether the association between priming and source memory can alternatively be explained as being driven by recognition or fluency. We first reproduced the basic priming-source association (Experiment 1). In Experiments 2 and 3, we found that the association persisted even when the task was modified so that overt and covert recognition judgements were precluded. In Experiment 4, the association was again present even though fluency (as measured by identification response time) could not influence the source decision, although the association was notably weaker. These findings suggest that the association between priming and source memory is not attributable to a contribution of recognition or fluency; instead, the findings are consistent with a single-system account in which a common memory signal drives responding.

Memory can be expressed in a variety of ways, such as a change in identification or detection of an item due to previous exposure to the item (long-term repetition priming) or the ability to determine whether an item had been encountered before in a particular context (recognition memory). Prominent theories explain these particular phenomena as being driven by distinct memory systems, signals or processes. Under some theoretical accounts, priming is driven by an implicit (unconscious or nondeclarative) memory system, whereas recognition memory is driven by a functionally and neurally distinct explicit (conscious or declarative) memory system (e.g., Squire, 1994, 2004, 2009; Squire & Dede, 2015; Tulving & Schacter, 1990). This multiple systems account of memory is pervasive in psychology textbooks as the default model of memory (e.g., Baddeley et al., 2014), and independent memory systems are still used to explain differential memory performance (e.g., Henson et al., 2016). Evidence for a multiple systems theory of memory is based on functional and neural dissociations between tasks (e.g., Craik et al., 1994; Jacoby & Dallas, 1981; Schacter et al., 2007; Squire, 2009; Staresina et al., 2011), though there is evidence challenging these findings and/or inferences (e.g., Addante, 2015; Berry et al., 2014; Buchner & Wippich, 2000; Dunn, 2003; Lukatela et al., 2007; Meier et al., 2009; Mulligan & Osborn, 2009; Ostergaard, 1992; Poldrack, 1996; Thakral et al., 2016).

The counterview to the multiple systems model of memory is that memory expression in different tasks, such as priming and recognition, is based on the same underlying memory signal. Under such an account, higher memory strength for an item should be simultaneously associated with greater priming and higher recognition memory. Berry et al. (2012) tested this account using a conjoint priming and recognition memory paradigm where, for each item at test, participants were asked to identify a word as it clarified over a mask (to provide a measure of priming) and give a recognition judgement on a scale of certain-new to certain-old. In line with a single-system model, they found that identification for items judged old was faster than that of items judged new; the priming effect, as measured across all studied items, was greater than the priming effect for items not recognised, and identification RTs (response times) tended to decrease as recognition confidence increased. This has since been replicated many times and confirmed in formal modelling (e.g., Berry et al., 2006, 2008a, 2008b, 2010; 2014; 2017; Mazancieux et al., 2019; Ward et al., 2013; see Shanks & Berry, 2012, for a review).

However, under some accounts of recognition memory, recognition memory itself is driven by two processes: recollection and familiarity (e.g., Yonelinas, 2002). While recollection relies on explicit retrieval of memory, familiarity is often argued to be driven by repetition priming (e.g., Jacoby & Dallas, 1981; Mandler, 1980). This means that the association of priming and recognition memory could be driven by this shared, implicit component and leaves the question of whether the same memory signal can drive performance in priming and a memory task that is traditionally seen as reliant on explicit memory.

In Lange et al.’s (2019) study, we therefore extended the behavioural and modelling work of Berry et al. (2012) to source memory. In source memory tasks, participants are asked to retrieve the exact context an item was studied in, such as whether it was shown in red or blue font, on the top or the bottom of the screen, or on a beach or woods background. These tasks cannot be solved by relying on familiarity but require the explicit retrieval of memorial information (but see Diana et al., 2008; Taylor & Henson, 2012). In this extended task, at study participants were shown words at the top or the bottom of the screen. At test, participants first identified an item as it clarified across a mask, then gave a recognition confidence rating, followed by a source confidence rating. We replicated findings of the association of priming and recognition memory and observed the analogous association of priming and source memory: items with correct source decisions tended to also have faster identification RTs (for similar findings using a recall task as the source memory task, see Mazancieux et al., 2019, Exp 1). These results are consistent with a single memory signal underlying responding where greater memory strength of an item is more likely associated with greater priming, correct “old” recognition judgements, and correct source judgements.

While the core assumption of a single memory signal or multiple independent memory signals is central to the predictions about the association of those memory tasks, auxiliary assumptions about the response mapping describes how responding in one task changes with responding in another. In the standard response mapping, responses are assumed to be made independently of one another. For the association of priming and source memory, for example, this means that the magnitude of the priming effect should monotonically increase from “sure-(incorrect source decision)” to “sure-(correct source decision).”^1^ However, in all Lange et al.’s (2019) experiments, priming tended to be highest at both end points of the rating scale and lowest at the mid-point of the scale. In other words, priming increased with increasing confidence in the source decision, regardless of whether that decision was correct or incorrect.

Given source memory ratings followed recognition ratings in our task, we considered whether this unexpected pattern in the association of priming and source memory was due to the recognition ratings that preceded the source confidence ratings, that is, that recognition and source memory responses were not made independently. It is well-established that there is some dependency between source- and recognition-ratings, such that source decisions made with high confidence are more likely when recognition decisions are made with high confidence (e.g., Hautus et al., 2008; Starns et al., 2013) and that this is a consequence of more than just a shared memory signal (Starns & Ksander, 2016). Models of recognition and source memory incorporate this by allowing source decision criteria or response mapping to change with the recognition rating (e.g., Hautus et al., 2008; Klauer & Kellen, 2010; Onyper et al., 2010). When we adapted the response mapping to include the dependency between these responses, the single-system model of our conjoint memory tasks captured the finding that correct source decisions are associated with greater priming than incorrect source decisions overall, and that priming increases with source confidence regardless of whether the source response was correct.

One possibility is that the better prediction of the model with changed response mapping is evidence that the underlying process that gives rise to the specific characteristics of the association between priming and source memory is the decisional dependence of source memory ratings on preceding recognition memory ratings. In this article, we sought to test this empirically. If source memory confidence ratings change with recognition confidence ratings, removing recognition confidence ratings should remove that decisional bias. Then, overall, correct source decisions should still be associated with greater priming than incorrect source decisions (in line with the core assumption of the single-system model), but priming should now gradually increase with increasing confidence in the correct source decision. Experiment 1 is a replication of Experiment 2 by Lange et al. (2019) to re-establish the previously observed pattern of the association of priming and source memory. We then sought to determine whether the association would persist even when overt (Experiment 2) and covert (Experiment 3) recognition judgements were precluded. In Experiment 4, by measuring priming and source decisions in separate, rather than interleaved phases, we tested whether the priming-source association would persist under conditions where other factors, like the fluency of identification, would not influence the source decision.

## Experiment 1

### Method

#### Participants

Thirty six individuals (7 male; *M* age = 24.20, *SD* = 9.52) took part in the experiment for payment of £8. This sample size provided a power of 0.8 to detect a medium-sized effect in a repeated measures design with two levels (i.e., a Cohen’s *d*_z_ approximately equal to 0.48) based on calculations for a pilot study. We used the same sample size in each subsequent experiment. Participants in each experiment were recruited using a University of Plymouth participation pool. Ethics were approved by the University of Plymouth ethics board. All participants provided informed consent prior to participating in the experiment.

#### Materials

The stimulus pool consisted of 384 four-letter low-frequency words, selected from the Medical Research Council psycholinguistic database (Coltheart, 1981). The frequency of occurrence ranged from 1 to 13 per million, and there were no concreteness or imageability constraints. Archaic and colloquial terms were excluded. For each participant, 176 words were randomly assigned to be the old stimuli, another 176 words were selected to be the new stimuli, and a further 32 words were selected to be the stimuli appearing on primacy and recency buffer trials in the study phase.

#### Procedure

At the beginning of the experiment, participants completed six practice trials of the continuous identification task (CID; Berry et al., 2012; Feustel et al., 1983; Lange et al., 2019; Stark & McClelland, 2000) to familiarise themselves with the task prior to the experimental trials. The CID procedure was the same as that of Lange et al. (2019). On each CID trial a single word was flashed for longer and longer durations, becoming clearer over time. Participants were instructed to press the Enter key as soon as they were sure that they could identify the word correctly. Accuracy and speed were emphasised in the task instructions. At the start of each trial, a fixation mask “####” was presented in 24-point Courier font for 1,000 ms. Next, the word was presented in 20-point Courier font for 16.7 ms (one screen refresh at 60 Hz). The mask was then presented for 233.3 ms, forming a 250 ms presentation block. There were thirty 250 ms presentation blocks. The stimulus duration increased by 16.7 ms on each alternate block, and the mask was always presented for the remainder of the 250 ms block. Thus, each CID trial was potentially 7,500 ms long, but could be terminated prematurely by the participant pressing the Enter key. When the Enter key was pressed, the mask was then re-presented for 16.7 ms. Next, a white outlined box was presented that indicated to the participant that he or she must type the word on the keyboard. Key presses were displayed in the box. Participants were told to press Enter after typing the word to advance to the next trial.

##### Study phase

Participants were told that they would see words presented below or above the centre of the screen for a brief duration and that their task was to remember the location of each word for a later test. Participants completed eight study-test blocks, which were identical except that the stimuli in each block were unique. At the start of each study block a “+”-fixation was presented for 500 ms in the centre of the screen. The words were presented for 2 s each, with half of them presented 0.9 cm below the central fixation point (i.e., subtending a vertical visual angle of approximately 0.69°, from a viewing distance of approximately 75 cm) and the other half 0.9 cm above the fixation point. The inter-stimulus interval was 100 ms. The assignment of words to the location and the order of presentation was randomised across participants. Participants completed 26 study trials per block, with the first and last two trials in each block designated as primacy and recency buffer trials. The buffer stimuli were not presented in the experiment again.

##### Test phase

Next, instructions were presented for the first CID-RS (i.e., CID with Recognition and Source judgements) test phase. Participants were told that they would again complete identification trials, and that some of the words were from the previous study block and some were novel. They were told that they must decide whether they thought the word was new (i.e., not shown previously) or old (i.e., studied) after each identification, and to indicate whether it was previously shown at the bottom or the top of the screen. They were informed to make that location judgement even for items they indicated were new and to guess if unsure. Participants were told that half of the words would be new and half would be old, and that half of the old words were presented at the bottom of the screen and half were presented at the top. There were 44 trials in each test block, composed of 22 old and 22 new items. On each trial, a word was presented in the centre of the screen using the same CID procedure as in the practice trials. After participants made their identification, the word was replaced by a recognition probe (“Is the word New or Old?”) and a rating scale (“1 = sure new, 2 = probably new, 3 = guess new, 4 = guess old, 5 = probably old, 6 = sure old”). After participants made their recognition judgement, a source memory probe was presented (“Was the word presented at the bottom or top?”) with a rating scale (“1 = sure bottom, 2 = probably bottom, 3 = guess bottom, 4 = guess top, 5 = probably top, 6 = sure top”). Participants used the number keys 1 through 6 on the main part of a QWERTY keyboard for the recognition judgements and the number keys on the number pad for the source memory judgement. Stickers were added to the number pad with up arrows indicating the “top” response and down arrows indicating the “bottom” response. After making their source memory judgement, a prompt was presented instructing participants to press the Enter key to start the next trial. On completion of the test block, participants were presented with the next study block. On completion of the final test block, the experiment terminated.

#### Initial screening of identification trials

In this experiment and subsequent ones, a trial was not included in the analysis if a word was misidentified during the identification phase of a trial or identification responses were too fast or too slow. Identification responses were corrected for minor typographical errors (e.g., where a number or a symbol was typed after the correctly typed word). One participant was excluded at this stage because they did not attempt to identify any words in the first study-test block. Overall, the proportion of misidentified trials after correction for typographical errors was low (*M* = 3.05%, *SD* = 2.58), as was the proportion of trials on which participants did not provide a response (*M* = 0.19%, *SD* = 0.78). The proportion of trials on which the identification RT was less than 200 ms or greater than three standard deviations above the mean identification RT (within participant) was also low (*M* = 1.22% of trials, *SD* = 0.49). Following Lange et al. (2019), these four types of trials were not analysed further. This left a sufficient number of valid trials for all individuals (*M* = 95.54, *SD* = 2.52, Min = 88.07%).

#### Measures

All analyses were conducted in *R* (R Core Team, 2019). For all relevant statistical comparisons, we excluded participants listwise if they had missing data in any cell of that analysis. Analysis of variance (ANOVAs) were calculated using *aov_car* function in the *afex* package (Singmann et al., 2020), with post hoc contrasts calculated with *emmeans* (Lenth, 2020). Degrees of freedom were corrected for violation of sphericity where necessary using the Greenhouse-Geisser correction. An alpha level of .05 was used for all statistical analyses and all *t*-tests were two-tailed. We also conducted equivalent Bayesian analyses, and report Bayes factors (*BF*) for all reported frequentist tests, using the *BayesFactor* package (Morey & Rouder, 2018), with the package’s default priors for all tests. We report the following effect sizes: η_P_^2^ for ANOVAs, Cohen’s *d_z_* (*d_z_*; mean difference of two dependent measures, divided by the average standard deviation of the difference of the two measures) for *t* tests. Trials were aggregated across study-test blocks for all analyses.

The priming effect was calculated as the mean identification RT for new items minus the mean identification RT for old items. Recognition discrimination was measured with *d′* (henceforth referred to as *recognition d′*), which is calculated as *z*(*p*[“old”| old])—*z*(*p*[“old”| new]), where *p*(“old”| old) = (number of hits + 0.5)/(number of old items + 1) and *p*(“old”| new) = (number of false alarms + 0.5)/(number of new items + 1), following Snodgrass and Corwin (1988). The pattern of results for *P_r_*, which is the measure of discriminability in the two-high threshold model and is calculated as *p*(“old”| old)—*p*(“old”| new), was the same, so we only report recognition *d′* throughout. Recognition response bias was measured with *c* (henceforth referred to as *recognition c*), which is calculated as −0.5 * (*z*(*p*[“old”| old]) + *z*(*p*[“old”| new])). Source discrimination was measured with *d*′ (henceforth referred to as *source d′*). For this measure, source-top items were arbitrarily designated as targets and source-bottom items as nontargets; thus, source *d′* = *z*(*p*[“top”| top])—*z*(*p*[“top”| bottom]), where *p*(“top”| top) = (number of correct top responses + 0.5)/(number of source-top items + 1) and *p*(“top”| bottom) = (number of incorrect top responses + 0.5)/(number of source-bottom items + 1). The pattern of results for source accuracy—calculated as (number of “top”|top items + number of “bottom”|bottom items)/number of old items—was the same, so only the former is reported. Source bias was measured with *c* (henceforth referred to as *source c*) and calculated as −0.5* ( *z*(*p*[“top”| top]) + *z*(*p*[“top”| bottom])).

For the analysis of identification RTs classified according to source confidence ratings, responses were collapsed across source-top and source-bottom items. Source ratings 3, 2, and 1 for source-bottom items and 4, 5, and 6 for source-top items constituted correct source decisions with increasing certainty of response, while source ratings 4, 5, and 6 for source-bottom items and 3, 2, and 1 for source-top items constituted incorrect source decisions.

##### Reliability of measures

Prior research has shown that it is important to consider the relative reliabilities of direct and indirect memory tasks when comparing task performance (Buchner & Wippich, 2000). Accordingly, split-half correlations were used to determine the reliability of the priming, recognition, and source measures in all experiments. To calculate these, we first split the data from each participant into odd- and even-numbered trials and then calculated the priming effect, recognition *d*′, and source *d*′ in each half. The split-half correlations were then given as the Pearson correlation between performance in each half across participants. In Experiment 1, these were large and significant, priming, *r*(33) = .90, *p* < .001, *BF* = 1.94 × 10^9^; recognition *d′*, *r*(33) = .90, *p* < .001, *BF* = 3.55 × 10^9^; source *d′*, *r*(33) = .81, *p* < .001, *BF* = 7.70 × 10^5^.

### Results

Considering first overall levels of memory performance, the priming effect, recognition *d′* and source *d′* all exceeded chance (0): *M* priming = 247 ms, *SE* = 34, *t*(34) = 7.17, *p* < .001, *d* = 1.22, *BF* = 5.11 × 10^5^; *M* recognition *d′* = 1.23, *SE* = 0.10, *t*(34) = 12.02, *p* < .001, *d* = 2.03, *BF* = 8.61 × 10^10^; *M* source *d′* = 0.80, *SE* = 0.11, *t*(34) = 7.48, *p* < .001, *d* = 1.26, *BF* = 1.16 × 10^6^. Table 1 shows the mean identification RT for new and old items, and also the mean hit rate and false alarm rate for recognition and source decisions. Neither recognition nor source responding was biased overall (recognition *c* = −0.04, *SE* = 0.04, *t*(34) = 0.98, *p* = .33, *d* = 0.17, BF = 0.28; source *c* = 0.01, *SE* = 0.05, *t*(34) = 0.11, *p* = .91, *d* = 0.02, *BF* = 0.18).

**Table 1.** Mean Identification RTs for New and Old Items Across Experiments and Mean Hit and False Alarm Rates for the Source Memory Tasks.

	Identification RT (ms)			Source		
	Old items	New items	Priming effect	Hit	False alarm	*d′*
Experiment 1						
*M*	1,942	2,188	247	0.64	0.36	0.88
*SE*	62	77	34	0.02	0.02	0.15
Experiment 2						
*M*	2,029	2,277	248	0.65	0.35	0.88
*SE*	86	97	30	0.02	0.02	0.15
Experiment 3						
*M*	1,930	–	–	0.73	0.27	1.34
*SE*	82			0.02	0.02	0.15
Experiment 4						
*M*	1,678	–	–	0.65	0.35	0.81
*SE*	64			0.02	0.02	0.13

RT: response time; *SE*: standard error.

There was evidence for correlations between these overall measures, though this was only substantial for the association of recognition and source memory (priming and recognition *d′*, *r*(34) = .35, *p* = .041, *BF* = 2.32; priming and source *d′*, *r*(34) = .33, *p* = .056, *BF* = 1.84; recognition *d′* and source *d′*, *r*(34) = .82, *p* < .001, BF = 1.82 × 10^6^).

As in Lange et al.’s (2019) study, we expected associations between priming and source memory to be evident when broken down according to the source decision. We consider two aspects of the data: (a) the difference in the magnitude of the priming effect for items with correct and incorrect source decisions, and (b) how the priming effect varies with participants’ confidence in their source decision.

**Figure 1.**
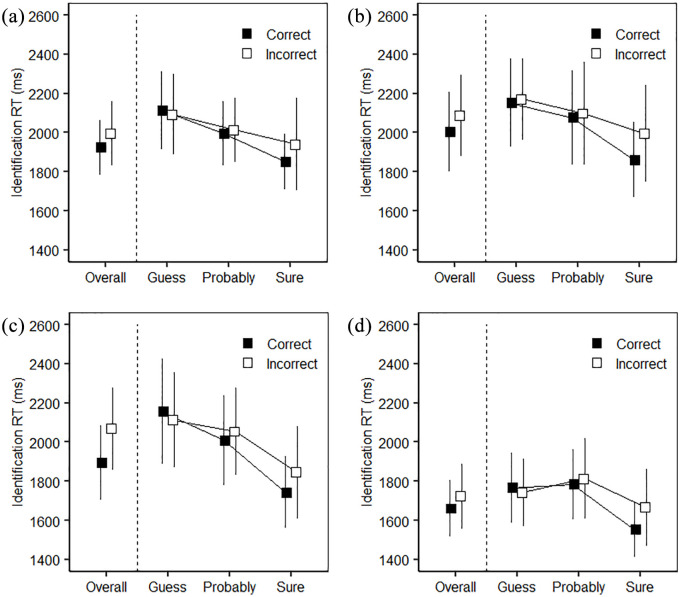
Association of source confidence and identification RT in (a) Experiment 1, (b) Experiment 2, (c) Experiment 3, and (d) Experiment 4. Error bars indicate the 95% confidence interval.

First, the priming effect for items with correct source decisions was significantly greater than for items with incorrect source decisions (*M* difference = 71 ms, *SE* = 24), *t*(34) = 3.00, *p* < .005, *d* = 0.51, *BF* = 7.76), see the left-hand side of Figure 1a). This difference was consistent across individuals, being present in 69% of participants.

Second, we examined identification RTs for correct and incorrect source decisions across participants’ confidence. This analysis is limited to studied items, i.e., items that can be associated with a correct and incorrect source decision. Table 2 shows the mean number of items at each level of this analysis. Please see the supplementary material for the analysis of the relationship of Identification RT and source confidence for new items. Identification RTs tended to decrease (i.e., the priming effect was greater) as confidence in the source decision increased, as is shown in the right-hand side of Figure 1a. This trend was confirmed in a 3 (source confidence: guess, probably, sure) × 2 (source decision: correct, incorrect) repeated measures ANOVA, which yielded a significant main effect of source confidence, *F*(1.63, 48.77) = 11.62, *MSE* = 70,424, *p* < .001, η_P_^2^ = .28, *BF* = 9.79 × 10^2^. Four participants could not be included in this ANOVA because they had zero responses for particular cells of the analysis (hence *N* = 31 for this analysis). Post hoc analyses confirmed a significant linear trend, *t*(43) = 4.82, *p* < .001, with higher-level trends not significant (*p* > .89). Source decisions given a high confidence rating were associated with a faster identification than source decisions given a low confidence rating, *p* < .001 (the remaining comparisons, Bonferroni-adjusted, *p* > .043). There was no main effect of source decision, *F*(1, 30) = 1.14, *MSE* = 32431, *p* = .29, η_P_^2^ = .04, *BF* = 0.22, or interaction, *F*(2, 60) = 1.16, *MSE* = 40521, *p* = .32, η_P_^2^ = .04, *BF* = 0.23.

**Table 2.** Mean Number of Items with Correct and Incorrect Source Decisions Assigned Low-Medium-High Confidence Ratings.

	Correct source decision			Incorrect source decision		
	Low	Medium	High	Low	Medium	High
Experiment 1 (*N* = 31)						
*M*	27.26	31.10	48.45	25.26	26.45	11.03
*SE*	2.91	2.57	4.75	2.48	2.48	1.52
Experiment 2 (*N* = 30)						
*M*	30.90	25.83	51.37	28.63	19.17	11.17
*SE*	16.86	9.38	31.33	15.23	10.37	8.90
Experiment 3 (*N* = 33)						
*M*	21.67	25.58	78.54	18.39	16.42	9.33
*SE*	1.72	2.17	6.75	1.86	1.87	1.62
Experiment 4 (*N* = 30)						
*M*	28.00	29.03	49.83	26.87	20.57	9.80
*SE*	2.61	2.62	6.54	2.56	2.09	1.85

*SE*: standard error.

Briefly, in this experiment, we also replicated the association of priming and recognition memory shown by Berry et al. (2012) and Lange et al. (2019). For old items, identification was faster for items judged old than items judged new, *M* difference = 210 ms, *SE* = 51, *t*(34) = 4.15, *p* < .001, *dz* = 0.70, *BF* **=** 127, and identification RTs decreased with increasing recognition confidence (*p* < .001, though *p* < .015 for quadratic and cubic trends). For new items, there was no clear evidence for an effect of fluency, that is, *M* difference in identification RT to new items judged old and new = 48 ms, *SE* = 25, *t*(34) = 1.95, *p* = .060, *dz* = 0.33, *BF* **=** 0.98, though overall identification RTs decreased with increasing recognition confidence (*p* < .001, all higher-level contrasts: *p* > .050).

### Discussion

These results are consistent with those of Lange et al. (2019), showing greater priming for correct than incorrect source decisions, and greater priming with increasing confidence regardless of the source decision. We also replicated the now well-established association of priming and recognition memory in this paradigm (e.g., Berry et al., 2012). Having established the association between priming and source, we now turn to test if recognition confidence ratings are central to the nature of this association. This is the theoretical assumption underlying the adapted response mapping in the single-system model by Lange et al. (2019). In all following experiments, we will not elicit overt recognition ratings from participants. In addition, in Experiments 3 and 4, we will also limit covert recognition judgements, that is, judgements of an item’s oldness in the absence of an instruction to do so, by only showing old items at test.

## Experiment 2

In Experiment 2, we removed the requirement to make overt recognition judgements at test. To be clear, under a single-system account of performance in both tasks, the association between priming and source memory is predicted to persist even when recognition confidence ratings are not made. Thus, identification RTs for items with correct source decisions should still tend to be shorter than those of items with incorrect source decisions. However, if the nature of the association is driven by the recognition confidence ratings preceding source memory judgements, as formally modelled by Lange et al. (2019), we expect that the removal of recognition confidence judgements from the test phase will eliminate this influence. Then identification RTs should monotonically decrease from certain incorrect to certain correct source judgements, consistent with a single-system account with linear decision bounds (i.e., a standard response mapping in which source ratings are independent of recognition ratings).

### Method

#### Participants

Thirty six individuals (10 male; *M* age = 23.00, *SD* = 4.11) took part in the experiment for payment of £8.

#### Materials and procedure

Materials and procedure were identical to Experiment 1, bar the following changes. Critically, we removed recognition judgements from the test phase. At test, for each trial participants were first asked to identify a word in the identification task, followed by judging whether it had been presented at the top or the bottom of the screen. In the instructions, participants were still told that half the items at test were old and half were new, and that half the old items had been presented at top and half at the bottom of the screen. In contrast to Experiment 1, participants gave their source ratings using the number keys in the main part of a QWERTY keyboard (as in the experiments by Lange et al., 2019).

#### Initial screening of identification trials and reliability of measures

The number of valid trials was on average *M* = 93.97% (*SD* = 3.51, Min = 84.38) after excluding trials with no identification response (*M* = 0.03%, *SD* = 0.09), misidentified trials (*M* = 4.68%, *SD* = 3.38) and trials with identification RTs less than 200 ms or greater than three standard deviations above mean identification RT (*M* = 1.32%, *SD* = 0.62). Split-half correlations indicated that both priming, *r*(34) = .72, *p* < .001, *BF* = 1.21 × 10^4^, and source *d′*, *r*(34) = .88, *p* < .001, *BF* = 7.44 × 10^8^, were reliable.

### Results

The priming effect and source *d′* exceeded chance (0): *M* priming = 248 ms, *SE* = 30, *t*(35) = 8.33, *p* < .001, *d* = 1.39, *BF* = 1.40 × 10^7^; *M* source *d′* = 0.88, *SE* = 0.15, *t*(35) = 6.02, *p* < .001, *d* = 1.00, *BF* = 2.34 × 10^4^. Table 1 shows the mean identification RT for new and old items, and also the mean hit rate and false alarm rate for source decisions. Source responding was not biased overall (source *c* = 0.03, *SE* = 0.03), *t*(35) = 0.90, *p* = .37, *d* = 0.15, *BF* = 0.26. Across participants, the priming effect (in ms) was significantly correlated with source *d′*, *r*(34) = .39, *p* = .018, *BF* = 4.24.

As in Experiment 1, we are concerned with the association of priming and source memory when broken down according to the type of response. First, the priming effect for items with correct source decisions was significantly greater than for items with incorrect source decisions (*M* difference = 82 ms, *SE* = 21), *t*(35) = 3.82, *p* < .001, *d* = 0.64, *BF* = 55.68 (see the left-hand side of Figure 1a). This difference was present in 72% of participants. Second, the 3 (source confidence: guess, probably, sure) × 2 (source decision: correct, incorrect) repeated measures ANOVA showed a main effect of source confidence, *F*(1.49, 43.18) = 11.86, *MSE* = 95,936, *p* < .001, η_P_^2^ = .29, *BF* = 2.16 × 10^3^. Six participants could not be included in this ANOVA because they had zero responses for particular cells of the analysis (hence *N* = 30 for this analysis). Post hoc analyses confirmed a significant linear trend, *t*(58) = 4.74, *p* < .001, with higher-level trends not significant (*p* > .32). Indeed, source decisions given a high confidence rating were associated with a faster identification than source decisions given a medium confidence, *p* = .006, or low confidence rating, *p* < .001. The main effect of source decision was not significant, *F*(1, 29) = 2.26, *MSE* = 67,176, *p* = .14, η_P_^2^ = .07, *BF* = 0.40. The Source Confidence × Source Decision interaction was absent, *F*(1.54, 44.57) = 1.06, *MSE* = 77951, *p* = .34, η_P_^2^ = .04, *BF* = 0.22.

### Discussion

The association between priming and source memory did not change with the removal of recognition confidence ratings. As expected, correct source decisions were associated with a greater magnitude of priming than incorrect source decisions. However, we expected that if the qualitative pattern of the association of priming and source memory across source confidence is affected by recognition confidence ratings, as implied by the adapted response mapping, then this pattern should change if participants do not give recognition confidence ratings. This was not the case. It is possible that this is because participants still engaged in a process of recognition for each item despite not being required to. Given that participants are presented with some old and some new items at test, they may have still assessed the overall strength of the item before making their source decision (i.e., made a covert recognition assessment). Accordingly, Experiment 3 repeated Experiment 2 except that only studied items were presented at test and participants were told this fact, removing the need for them to make any covert recognition judgement at test.

## Experiment 3

### Method

#### Participants

Thirty six individuals (5 male; *M* age = 21.67, *SD* = 5.12) took part in the experiment for partial course credit. Participants in each experiment were recruited using the University of Plymouth participation pool.

#### Materials and procedure

The procedure was identical to Experiment 2, bar one change. At test, participants were only shown items from the study phase, that is, no new items were presented. During the instructions of the test phase, they were informed that they would only see old items at test. For eight study-test blocks, participants were therefore shown 22 old items at test. The remainder of instructions and procedure remained the same. For every word shown at test, they first identified the word in a CID task (providing identification RT) and subsequently rated how confident they were that the word had been shown at the top or bottom of the screen on a six-point rating scale using the number 1 through 6 on the main part of a QWERTY keyboard.

#### Initial screening of identification trials and reliability of measures

The number of valid trials was on average *M* = 96.37% (*SD* = 2.51, Min = 86.93) after excluding trials with no identification response (*M* = 0.02%, *SD* = 0.09), misidentified trials (*M* = 2.32%, *SD* = 2.46) and trials with identification RTs less than 200 ms or greater than three standard deviations above mean identification RT (*M* = 1.29%, *SD* = 6.87). Since only old items were presented at test, we cannot calculate an overall priming effect as a contrast of identification RT for old and new items. Source memory, as measured by source *d′*, was reliable, as indicated by the split-half correlation, *r*(34) = .89, *p* < .001, *BF* = 1.42 × 10^9^.

### Results

Overall, source *d′* exceeded chance (0) (*M* source *d′* = 1.36, *SE* = 0.15), *t*(35) = 8.84, *p* < .001, *d* = 1.47, *BF* = 5.46 × 10^7^. Table 1 shows the mean identification RT for old items, and also the mean hit rate and false alarm rate for source decisions. Source responding was not biased overall (source *c* = 0.01, *SE* = 0.02), *t*(35) = 0.32, *p* = .75, *d* = 0.05, *BF* = 0.19.

Correct source decisions were associated with a faster identification than incorrect source decisions (*M* difference = 173 ms, *SE* = 30), *t*(35) = 5.76, *p* < .001, *d* = 0.96, *BF* = 1.12 × 10^4^ (see the left-hand side of Figure 1b). This difference was consistent across individuals, being present in 86% of participants. Regarding identification RTs for correct and incorrect source decisions across participants’ confidence, the pattern in the 3 (source confidence: guess, probably, sure) × 2 (source decision: correct, incorrect) repeated measures ANOVA was the same as in previous experiments. Three participants could not be included in this ANOVA because they had zero responses for particular cells of the analysis (hence *N* = 33 for this analysis). There was a significant main effect of source confidence, *F*(1.65, 52.71) = 44.37, *MSE* = 54,985, *p* < .001, η_P_^2^ = .58, *BF* = 7.22 × 10^15^. Post-hoc analyses confirmed a significant linear trend, *t*(64) = 9.18, *p* < .001, with the quadratic trend also significant (*p* = .04). Source decisions given a high confidence rating were associated with a faster identification than source decisions given a medium or low confidence ratings, *p*s < .001, and source decisions given a medium confidence rating were associated with faster identification than source decisions given a low confidence rating, *p* < .001. The main effect of source decision was not significant, *F*(1, 32) = 1.89, *MSE* = 30,833, *p* = .18, η_P_^2^ = .06, *BF* = 0.24, neither was the Source Confidence × Source Decision interaction, *F*(2, 64) = 3.07, *MSE* = 28,641, *p* = .053, η_P_^2^ = .09, *BF* = 0.64.

### Discussion

The pattern of results in Experiment 3 was near-identical to the one in Experiment 2. Overall, correct source decisions were associated with more priming than incorrect source decisions, and priming increased with source confidence regardless of the source decision. This pattern is consistent with a single-system model with the adapted response mapping. However, the adapted response mapping is based on recognition confidence ratings influencing source memory confidence ratings. With both overt and covert recognition judgements removed in Experiments 2 and 3, this process cannot be driving the observed association. One possibility is that the perceived speed of identification in the priming task affected source ratings. For example, in the absence of recognition confidence ratings, perceived fast identifications in the CID task may have resulted in more confident source decisions than perceived slow identifications. In Experiment 4, we investigate if the association of priming and source memory persists when the contribution from either recognition confidence judgements or the identification task is eliminated.

## Experiment 4

In Lange et al.’s (2019) study and Experiment 1, source memory judgements were preceded by recognition judgements. In Experiments 2 and 3, we eliminated overt and covert recognition judgements, leaving source memory judgements to be directly preceded by the identification task. Given the association of priming and source memory persisted when recognition ratings were absent, it is possible that the perceived speed of identification in the priming tasks directly preceding the source memory task could influence the manner in which priming and source memory are related. As in some experiments by Lange et al. (2019), we therefore separated the identification task from the source memory task. While the perceived speed of identification is unlikely to affect the “correctness” of a source decision, it could be responsible for the extremeness of the confidence rating (as seemed to be the case for identification and recognition ratings by Lange et al., 2019). To investigate this possibility, Experiment 4 repeated Experiment 3, but measured identification RTs and source decisions in separate phases, rather than in an interleaved manner on each trial.

### Method

#### Participants

Thirty six individuals (8 male; *M* age = 19.72, *SD* = 1.21) took part in the experiment for partial course credit. One participant was excluded for using their phone during the experiment.

#### Materials

The materials were identical to previous experiments.

#### Procedure

The procedure was identical to Experiment 3, bar one change. At test, the identification task and source memory rating task of the test phase were not interleaved. At the start of the test phase in each study-test block, participants first completed the identification task for all items. Following this, they gave source judgements on a 6-point scale for all items. Here, on each source rating trial, the item was presented above the source rating scale in the centre of the screen in Courier New font (i.e., the same font as used during the study phase). The remainder of instructions, procedure, and number of items remained the same.

#### Initial screening of identification trials

The number of valid trials was on average *M* = 93.43 % (*SD* = 3.58, Min = 82.95) after excluding misidentified trials (*M* = 1.43%, *SD* = 0.59) and trials with identification RTs less than 200 ms or greater than three standard deviations above mean identification RT (*M* = 1.43%, *SD* = 0.59).

#### Measures

Since only old items were presented at test, we cannot calculate an overall priming effect as a contrast of identification RT for old and new items. We report source memory as in previous experiments.

##### Reliability of measures

Source memory, as measured by source *d′*, was reliable, *r*(33) = .90, *p* < .001, BF = 5.5 × 10^9^.

### Results

Source *d′* exceeded chance (0) (*M* source *d′* = 0.81, *SE* = 0.13), *t*(34) = 6.33, *p* < .001, *d* = 1.07, *BF* = 4.93 × 10^4^. Table 1 shows the mean identification RT for old items, and also the mean hit rate and false alarm rate for source decisions. Source responding was not biased overall (source *c* = −0.01, *SE* = 0.02), *t*(34) = 0.56, *p* = .58, *d* = 0.09, *BF* = 0.21.

Correct source decisions were associated with a faster identification than incorrect source decisions (*M* difference = 62 ms, *SE* = 22), *t*(34) = 2.80, *p* < .001, *d* = 0.47, *BF* = 5.00 (see the left-hand side of Figure 1d). This difference was consistent across individuals, being present in 60% of participants.

Second, we examined identification RTs for correct and incorrect source decisions across participants’ confidence. Five participants could not be included in this analysis because they had zero responses for particular cells of the analysis (hence *N* = 30 for this analysis). Identification RTs tended to decrease (i.e., the priming effect was greater) as confidence in the source decision increased, as is shown in the right-hand side of Figure 1d, *F*(1.63, 47.40) = 15.97, *MSE* = 44,788, *p* < .001, η_P_^2^ = .36, *BF* = 6.42 × 10^4^. Post- hoc analyses confirmed a significant linear trend, *t*(58) = 4.13, *p* < .001, though the quadratic trend was also significant (*p* < .001). Source decisions given a high confidence rating were associated with a faster identification than source decisions given a medium or low confidence, *p*s < .001, while there was no significant difference between items given a low or medium confidence rating, *p* = .63, all comparisons Bonferroni-adjusted. There was no sufficient evidence for a main effect of source decision, *F*(1, 29) = 2.09, *MSE* = 32,788, *p* = .16, η_P_^2^ = .07, *BF* = 0.36, or a Source Confidence × Source Decision interaction, *F*(2, 58) = 2.43, *MSE* = 29,953, *p* = .097, η_P_^2^ = .08, *BF* = 0.58.

### Discussion

Overall, the results replicate previous experiments: correct source decisions are associated with more priming than incorrect source decisions, and priming increases as source confidence increases regardless of the source decision. However, in contrast to Experiment 3, which was identical to this experiment bar, the separation of identification task and source memory judgements, all effects are considerably weaker. There is a simple explanation for this overall weaker association. Taking two measurements of the same memory signal at distinct time points (where different sources of noise and forgetting can affect the measures) results in a poorer measure than taking the two measurements concurrently.

## General discussion

In Lange et al.’s (2019) study, we argued that the same memory signal underlies responding in priming, recognition, and source memory tasks, with a greater signal leading to higher performance in all three tasks. For the association of priming and source memory, a correct source decision (as a consequence of a greater memory signal, hence a greater source strength) was associated with faster identification in a priming task (as a consequence of a greater memory signal). While this association was graded, priming was associated with confident source judgements, regardless of whether they were correct (associated with high source strength) or incorrect (associated with low source strength). To account for this pattern in a formal signal detection model, we had to assume that source confidence ratings were not only driven by source strength, but also influenced by the preceding recognition confidence rating. That is, that source confidence judgements were not made independently of recognition confidence judgements. In this article, we examined the theoretical assumption underlying this modelling choice empirically.

After replicating the pattern of the association of priming and source memory (Experiment 1), removing overt and covert recognition ratings from the task (in Experiments 2 and 3, respectively) did not change the nature of the association of priming and source memory. This suggests that it is not recognition ratings per se that are driving the observed association, even though allowing for decisional dependence between recognition and source ratings in the single-system model of Lange et al. (2019) suggests this as the underlying process. However, in Experiments 2 and 3, source confidence judgements were not made in isolation: they were still preceded by the identification task. It is possible the particular decisional dependency of source on recognition ratings is merely one expression of a more generic fluency or decisional dependency mechanism.

In Experiment 4, we therefore tested if the perceived speed of identification could have simply acted in lieu of recognition confidence ratings in Experiments 2 and 3. We separated identification and source memory task into different test blocks (i.e., trials were no longer interleaved), and indeed the association of priming and source memory weakened. This is in line with findings by Lange et al.’s (2019) study where the association between priming and recognition weakened when this source of fluency was similarly eliminated. This is not to say that fluency did not affect source confidence judgements in Experiment 4. The pattern of the association still suggests that something other than source strength contributes to the source confidence judgements. It is possible, if unlikely, that participants are able to access their memory for the speed of identification of individual items directly when coming to assess their confidence in their source decision. Alternatively, participants’ assessment of an item’s source strength may still be influenced by their assessment of an item’s strength overall.

Overall our results suggest that implementing a dependency of source confidence judgements on recognition confidence judgements (e.g., Starns & Ksander, 2016) captures only one expression of decisional dependency between tasks. Across the work here and in Lange et al.’s (2019) study, we suggest that such dependencies are particularly present in interleaved memory tasks. Treating memory judgements in these tasks as decisionally independent, in formal modelling or when drawing inferences from the data, therefore ignores crucial mechanisms that contribute to the observed patterns of data.

What does this mean for a single-system model of memory? To be clear, our findings do not suggest that fluency alone, or even primarily, is sufficient to explain the associations between memory tasks. Even when this daisy-chain of decisional dependencies in a conjoint task such as ours is broken up by separating the tasks, the association of priming and source memory persists. It is clear that fluency, as perceived speed of identification, or the preceding judgement of memory strength can contribute to the source confidence judgements participants make. However, fluency cannot affect the source decision in favour of Source A or Source B and therefore cannot result in an accurate source decision; this can only be driven by source strength. Overall, the persistence of the association between priming and source memory is consistent with a single-system account of memory, where performance in different memory tasks is driven by the same memory strength signal as opposed to distinct, independent memory signals.

## Supplemental Material

sj-docx-1-qjp-10.1177_17470218211008406 – Supplemental material for Explaining the association between repetition priming and source memory: No evidence for a contribution of recognition or fluency Click here for additional data file. Supplemental material, sj-docx-1-qjp-10.1177_17470218211008406 for Explaining the association between repetition priming and source memory: No evidence for a contribution of recognition or fluency by Nicholas Lange and Christopher J Berry in Quarterly Journal of Experimental Psychology

## Notes

1. For items shown at the bottom of the screen that refers to judgements ranging from “sure-top” to “sure-bottom, and for items shown at the top of the screen from ‘sure-bottom” to “sure-top.”
