# Supplementary material for: Explaining the association between repetition priming and source memory: No evidence for a contribution of recognition or fluency
Source: Q J Exp Psychol (Hove). 2021 Apr 12;74(10):1806–17. doi: 10.1177/17470218211008406 (PMC8392247; doi:10.1177/17470218211008406)
Supplement: sj-docx-1-qjp-10.1177_17470218211008406 – Supplemental material for Explaining the association between repetition priming and source memory: No evidence for a contribution of recognition or fluency [file sj-docx-1-qjp-10.1177_17470218211008406.docx]

Supplementary Material for:

**Explaining the association between repetition priming and source memory: No evidence for a contribution of recognition or fluency**

Nicholas Lange and Christopher J. Berry

**Supplementary analysis**

for “Explaining the association between repetition priming and source memory: No evidence for a contribution of recognition or fluency”

In the manuscript, we only discussed the relationship of source ratings and identification RT for studied items. Here, we additionally analyse this relationship for new items presented at test in Experiment 1 and 2, see Table S1. This analysis cannot be done for Experiment 3 and 4 since new items were not presented.

**Experiment 1**

Identification RT decreased with increasing source confidence judgments for new items, F(1.46, 43.79) = 6.22, MSE = 81586, p = .009, pes = .17, BF = 10.36, with items given a high source confidence rating associated with significantly faster RTs than items given a low, p = .015, or medium source confidence rating, p = .007.

**Experiment 2**

There was no sufficient evidence that identification RT decreased with increasing source confidence for new items, F(2, 60) = 1.70, MSE = 31369, p = .19, pes = .05, BF = 0.36.

Table S1.

*Mean Number of New Items and Identification RT with Correct and Incorrect Source Decisions Assigned Low-Medium-High Confidence Ratings*

|  | Source Confidence | | |
| --- | --- | --- | --- |
|  | Low | Medium | High |
| Experiment 1 (N = 31) | | | |
| *Number* | 108.68 (6.14) | 49.19 (5.83) | 8.16 (1.57) |
| *RT* | 2206 (82) | 2221 (83) | 2025 (87) |
|  |  |  |  |
| Experiment 2 (N = 31) | | | |
| *Number* | 103.00 (7.02) | 49.71 (6.23) | 10.32 (1.87) |
| *RT* | 2277 (98) | 2287 (113) | 2211 (111) |
| *Note: SD in brackets* | | | |
